# Developmental variations in plasma leptin, leptin soluble receptor and their molar ratio in healthy infants

**DOI:** 10.1186/1475-2891-6-11

**Published:** 2007-06-04

**Authors:** Winston WK Koo, Mouhanad Hammami, Elaine M Hockman

**Affiliations:** 1 The Carman and Ann Adams Department of Pediatrics, Wayne State University and Hutzel Hospital, Wayne State University, Detroit, Michigan, USA; 2 Computing and Information Technology, Wayne State University, Detroit, Michigan, USA

## Abstract

**Background:**

Leptin and its soluble receptor (sOB-R) are important to regulation of body composition but there are no data on the developmental variations in these plasma variables and their relationship with body composition measurements,

**Methods:**

Weight, length, and body composition (bone, fat and lean mass) by dual energy absorptiometry, and plasma variables were measured in healthy infants at 2, 4, 8 and 12 months.

**Results:**

15 whites and 29 African Americans (21 males and 23 females) with mean birth weight 3357 +/- 45 (SEM) g and gestation of 39.3 +/- 0.17 weeks were studied. The overall Z score for weight, length and weight for length during the study were 0.00 +/- 0.15, -0.08 +/- 0.11 and 0.12 +/- 0.14 respectively. With increasing age, plasma leptin (1.0 to 18.2, median 5.5 ng/mL) and sOB-R:leptin molar ratio (10.1 to 247.4, median 59.9) were lowered (r = -0.47, p < 0.01; and r = -0.37, p < 0.05 respectively), best predicted by   weight Z score and percentage of fat mass, and higher in African American and female. Presence of body composition measurements eliminated the race and gender effect on the plasma variables. Plasma sOB-R (49.5 to 173.9, median 81.3 ng/mL) did not change significantly with age and was correlated and predicted only by body composition measurements.

**Conclusion:**

In healthy growing infants, plasma leptin but not sOB-R decreases with age. Gender, race and anthropometric measurements are additional physiological determinants predictive of plasma leptin and the receptor:ligand ratio. However, body composition is the only variable that can predict plasma leptin and its soluble receptor and the receptor: ligand ratio; and body composition measurements eliminated the race and gender effect on these plasma variables.

## Background

Hormonal responses from the gastrointestinal tract, brain and body tissues associated with dietary intake and regulation of nutritional status and body weight are well described in adults [1-3]. There are increasing data indicating that similar hormonal changes can occur in children and infants [4-9]. Understanding their relationship with body composition measurements as indicators of tissue accretion might shed light on the physiological basis to integrate nutrition support, growth and tissue accretion.

Concentration of circulating leptin, an adipocyte hormone, reflects the amount of energy stored in adipose tissue and is considered a marker of nutritional status [1-3]. Leptin is bound to the soluble leptin receptor (sOB-R) in the circulation, which modulates steady state leptin levels by preventing the hormone from degradation and clearance [10], and sOB-R:leptin ratio may be considered a marker of bioavailable leptin.

In the neonate, dramatic changes in circulating leptin and sOB-R with a decrease in leptin by as much as 80% [5-7] and increase in sOB-R by >5 folds [7] are reported. At birth, weight and body mass index are positively correlated with plasma leptin concentrations [5,6,8], and one report indicated that sOB-R is inversely related to birth weight [7]. This pattern of changes in leptin and its sOB-R and their relationship with body weight and body mass index presumably reflect the loss of placental contribution for leptin and the physiologic adaptation to lower availability of free leptin, thereby allowing an increase in energy intake to initiate the phase of rapid postnatal growth.

Infancy is the period of most rapid postnatal growth and one report in healthy but relatively malnourished infants with average weight for age Z score of -1.9 at 52 weeks shows a decrease in plasma leptin from birth to 1 year. Changes in plasma sOB-R in infants beyond the immediate newborn period are not well defined. Furthermore, the relationship between body composition measurements as reflection of tissue accretion, with these plasma variables is not known. The aim of this study is to define the developmental variations in circulating leptin and sOB-R in healthy infants specifically the physiologic determinants including age, gender, race, and measures of growth or body composition on these plasma variables.

## Methods

This is a cross sectional design with anthropometry, body composition and circulating leptin and sOB-R measurements performed on the same day for each subject. All subjects were singleton infants between 37 and 42 weeks gestation with appropriate birth weight for gestation [11] and studied at approximately 2, 4, 8 and 12 months. None of the infants had major malformation or medical or surgical conditions that may affect long term growth.

Weight, length, and head circumference were measured using standard methods [12,13]. Infants were weighed in the nude to the nearest 5 g using an electronic scale (Seca, Toledo, OH) that was calibrated daily. Length was measured in duplicate to the nearest 0.1 cm with the infant in a recumbent position using O'Leary Lengthboard ™ (Ellard Instruments Ltd, Seattle, WA).

Body composition is indicated by total body bone mass as bone mineral content, and fat and lean mass were determined by fan beam dual energy X-ray absorptiometry (DXA) (QDR 4500A, Hologic Inc, Waltham, MA). Scan acquisition techniques have been reported elsewhere [14]. Each infant was wrapped in cotton blanket for the scan. The use of a diaper with or without light undergarment for the infant was allowed prior to bundling the infant in the cotton blanket. However, all coverings were weighed with an electronic scale and the weight recorded. Scan analysis used software vKHS11 validated by carcass analysis [15,16]. Only scans with no significant movement artifacts [17] were included in data analysis. In our laboratory, the precision error [18] from duplicate infant whole body scans for bone, fat and lean mass were 2.6, 7.1 and 2.5% respectively. Bone, fat and lean mass also were expressed as a percent of DXA measured total body mass.

Plasma samples for the measurement of leptin and sOB-R were kept at -70°C until measurement. Plasma leptin and sOB-R were measured using the commercial enzyme linked immunoassay kits from the same manufacturer (Diagnostic Systems Laboratories, Inc., Webster, TX). Recombinant human leptin and human soluble leptin receptor were used as standards and controls in the respective assays. In our laboratory, the coefficient variation of the leptin assay was 9% and for the sOB-R assay was 5%.

Ethical approval for the study was obtained from the Institutional Review Board for Human Investigations at Wayne State University, Detroit, MI. Written informed consent was obtained from the parent of each infant.

### Statistical analysis

The absolute values of anthropometric measurements were normalized by expression as Z scores using the age and gender matched normative data from the National Center for Health Statistics [19]. The absolute values for bone, fat and lean mass were transformed to a percentage of total weight. Plasma sOB-R:leptin molar ratio was calculated according to the molecular mass of 130 kD for sOB-R and 16 kD for leptin. All statistical analysis included the use of absolute and transformed measurements.

Pearson's correlation was used to determine the relationship for each plasma parameter (leptin, sOB-R and sOB-R:leptin ratio) with each anthropometric and each DXA parameter. Analysis of covariance was used to determine the relation of gender, race and age to plasma measurements. Stepwise regression analysis was used to determine the relative contribution from each of the physiologic variables in the prediction of plasma leptin, sOB-R, and sOB-R:leptin ratio. By design, the use of absolute and percentage of body composition measurement was mutually exclusive in regression analysis, as well as the use of anthropometric and DXA measurements, since body weight is the sum of body composition measurements; and both weight and length are predictive of various aspects of body composition [20-22]. Neither age nor gender was entered as independent variables with any analysis using Z scores since the Z scores were standardized to age and gender.

All values are mean +/- SEM. Statistical tests were performed with SPSS Version 13.5 for Windows (SPSS Inc., Chicago, IL) at an adopted significance level of 0.05 and were two-tailed.

## Results

There were 44 infants with mean birth weight 3357 +/- 45 g and gestation of 39.3 +/- 0.17 weeks, with 15 whites and 29 African Americans, and 21 (7 white) males and 23 (8 white) females. Anthropometric and DXA measurements and blood collection were performed at 56 +/- 0.8, 112 +/- 1.0, 240 +/- 1.5, and 366 +/- 2.7 days. Age was positively correlated with all absolute values of anthropometric and DXA measurements (p < 0.01 for all comparisons). Weight for age Z score (WAZ) decreased (r = -0.36, p = 0.02) but recumbent length for age (HAZ) and weight for length (WHZ) Z scores were not significantly different with age. The overall WAZ, HAZ and WHZ were 0.00 +/- 0.15 and -0.08 +/- 0.11, and 0.12 +/- 0.14 respectively. Bone mass as a percentage of total mass was increased (r = 0.58, p < 0.01) but the percentages of fat and lean mass did not change significantly with age. The overall percentages for bone, fat and lean mass were 2.5 +/- 0.04, 26.3 +/- 0.92, and 71.2 +/- 0.93 respectively.

**Figure 1:**
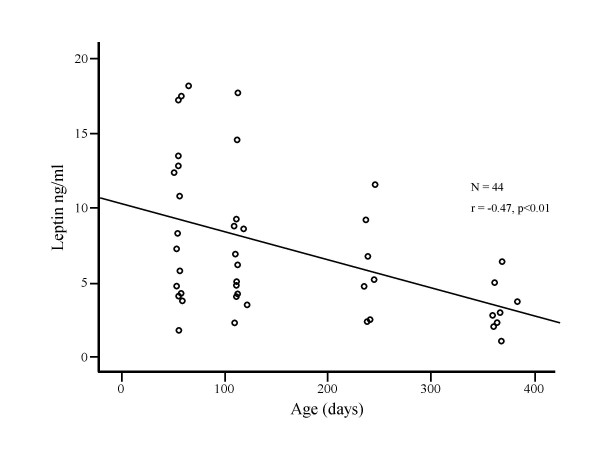
Variation in plasma leptin concentrations with age in healthy infants.

Plasma leptin concentrations varied from 1.0 to 18.2 (median 5.5) ng/mL and decreased with age (r = -0.47, p < 0.01) (Fig 1). The relationships between plasma leptin concentrations and anthropometric measurements are shown in Table 1. Z scores were better correlated with plasma leptin than absolute measurements. The relationships between plasma leptin concentrations and body composition measurements are shown in Table 2. Body compositions as percentage of total weight were better correlated with plasma leptin than absolute measurements. Percent fat mass was positively correlated with plasma leptin although both absolute and percent bone and lean masses were negatively correlated with plasma leptin. African American infants had higher plasma leptin concentrations (p < 0.05) after adjustment for age at study. There was no race and gender interaction effect on plasma leptin. Neither race nor gender affected absolute or percent fat mass.

**Table 1:** Correlation of plamsa leptin, leptin soluble receptor (sOB-R), and sOB-R:Leptin ratio with anthopometric measurements and their Z scores in term infants

	Leptin ng/mL	Leptin soluble receptor ng/mL	sOB-R:Leptin molar ratio
Absolute measurements			
Weight	-0.29	0.29	-0.14
Length	-0.44*	0.23	-0.30
Weight:length ratio	0.45*	-0.25	0.30
Z scores			
Weight	0.58*	0.22	0.66*
Length	0.05	0.29	0.21
Weight for length	0.57*	0.17	0.62*

Pearson correlation with 2 tailed significance: * p ≤ 0.01

**Table 2:** Correlation of plasma leptin, leptin soluble receptor (sOB-R), and sOB-R:Leptin ratio with body composition measurements as absolute values or as percentage of total weight in term infants

	Leptin ng/mL	Leptin soluble receptor ng/mL	sOB-R:Leptin molar ratio
Absolute measurements			
Bone	-0.32†	0.20	-0.21
Lean	-0.43*	0.17	-0.34†
Fat	0.16	0.47*	0.38†
As percentage of total weight			
Bone	-0.33†	-0.16	-0.33†
Lean	-0.50*	-0.33†	-0.66*
Fat	0.52*	0.31	0.66*

Pearson correlation with 2 tailed significance: * p ≤ 0.01, † p ≤ 0.05

**Figure 2:**
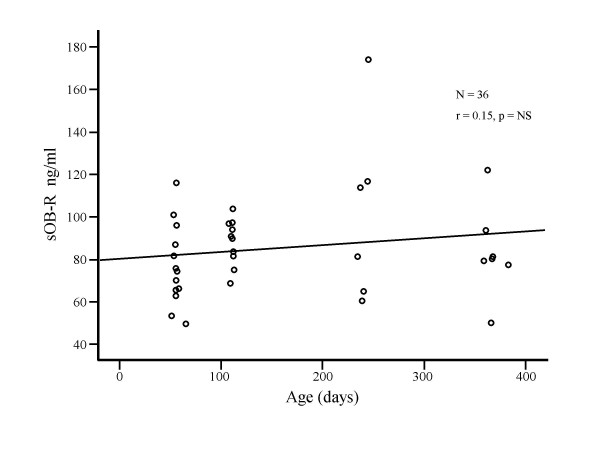
Variation in plasma leptin soluble receptor (sOB-R) concentrations with age in healthy infants.

Plasma sOB-R concentrations varied from 49.5 to 173.9 (median 81.3) ng/mL, did not change significantly with age (Fig 2) and were not significantly correlated with plasma leptin concentrations. There was no correlation between plasma sOB-R with any anthropometric measurements (Table 1). Plasma sOB-R was positively correlated with fat mass and negatively correlated with percent lean mass (Table 2). Plasma sOB-R was not affected by race or gender.

Plasma sOB-R:leptin molar ratio varied from 10.1 to 247.4 (median 59.9) and decreased with age (r = -0.37, p = 0.05). Weight and WAZ were the only anthropometric variables significantly correlated with plasma sOB-R:leptin ratio. Whereas, plasma sOB-R:leptin ratio was positively correlated with absolute and percent fat mass, but negatively correlated with lean mass and percent lean and bone mass. The relationship was stronger based on percent lean or fat mass than the absolute mass (Table 2). There was no race or gender effect on plasma sOB-R:leptin ratio.

Weight or WAZ had positive and length or HAZ had negative predictive effect on plasma leptin. Females have higher plasma leptin concentrations and sOB-R:leptin ratio compared to males. None of the physiologic variables entered into analysis with anthropometric measurements were predictive for plasma soluble receptor concentration (Table 3).

**Table 3:** Prediction values of physiologic variables including age, race, gender, and absolute values (left 2 columns) or age and gender specific Z scores (right 2 columns) of anthropometric measurements for plasma concentrations of leptin and leptin soluble receptor*, and leptin soluble receptor:leptin molar ratio.

Dependent variable – plasma leptin (ng/mL)			
Independent variables: R^2 ^0.49	Beta (p)	Independent variables: R^2 ^0.46	Beta (p)
Weight (g)	1.87 (0.001)	Weight Z score	0.83 (0.001)
Length (cm)	-2.17 (0.001)	Length Z score	-0.44 (0.01)
Gender (Female = 1, male = 0)	0.27 (0.03)		
			
Dependent variable – plasma soluble leptin receptor : leptin molar ratio			
Independent variables: R^2 ^0.50		Independent variables: R^2 ^0.44	
Age (d)	-0.75 (0.001)	Weight Z score	0.66 (0.001)
Weight (g)	1.51 (0.001)		
Gender (Female = 1, male = 0)	0.39 (0.01)		

* None of the physiologic variables was predictive for plasma soluble receptor concentration.

The absolute or percent fat mass was consistently predictive of plasma leptin, sOB-R and sOB-R:leptin molar ratio (Table 4). Percentage of body composition component was slightly better than the absolute values in the prediction of the plasma variables. Age was predictive of plasma leptin and sOB-R:leptin ratio. Presence of body composition measurements eliminated any race or gender effect on plasma sOB-R and sOB-R:leptin ratio.

**Table 4:** Prediction values of physiologic variables including age, race, gender, and absolute values (left 2 columns) or percent bone, lean and fat mass (right 2 columns) for plasma leptin and leptin soluble receptor concentrations, and leptin soluble receptor:leptin molar ratio

Dependent variable – plasma leptin (ng/mL)			
Independent variables: R^2 ^0.37	Beta (p)	Independent variables: R^2 ^0.37	Beta (p)
Age (d)	-0.65 (0.001)	Age (d)	-0.34 (0.01)
Fat (g)	0.44 (0.003)	Fat%	0.42 (0.003)
			
Dependent variable – plasma soluble leptin receptor (ng/mL)			
Independent variables: R^2 ^0.22		Independent variables: R^2 ^0.43	
Fat (g)	0.47 (0.004)	BMC%	-0.63 (0.004)
		Lean%	-14.1 (0.001)
		Fat%	-13.8 (0.001)
			
Dependent variable – plasma soluble leptin receptor : leptin ratio			
Independent variables: R^2 ^0.54		Independent variables: R^2 ^0.59	
Age (d)	-0.71 (0.001)	BMC%	-0.59 (0.002)
Fat (g)	0.72 (0.001)	Lean%	-7.04 (0.02)
		Fat%	-6.42 (0.03)

## Discussion

Study of leptin and its regulation has demonstrated its importance as an integral part of homeostatic mechanism in the regulation of body weight [1-3]. However, it is not known whether this is applicable to all life stages and what changes it may have during growth in which weight gain and tissue accretion rather than maintenance of body weight is the physiologic norm.

To our knowledge, this is the first report of the relationship of the circulating leptin and sOB-R concentrations to various physiological variables of growth and body composition specifically, bone, fat and lean masses, during infancy. Age appears to be a major physiologic determinant of plasma leptin concentrations. It is decreased during infancy in those with normal age and gender specific weight and length Z scores as indicated by our data, and is also decreased in infants with poor postnatal growth during longitudinal measurement of plasma leptin from cord blood and at 8, 16 and 52 weeks [9]. In this study, the initial measurement of circulating leptin and its soluble receptor at 2 months likely eliminated the confounding factors of placental leptin [23], the initial physiologic adaptation that occurs commonly with other endocrine systems [24], and the apparent transient increase in plasma leptin during the first weeks after birth observed by some [25,26] but not by other [9] investigators. In the two reports on postnatal increase in plasma leptin concentration, one reported an increase in plasma leptin at 30 days which was significantly correlated with interval weight gain [25] but no data was available beyond 30 days. In the other report, plasma leptin concentration was higher in term versus preterm infants up to 30 days but there was no significant difference between groups at 90 days. The increase in plasma leptin was correlated with weight gain and increase in subcutaneous tissue [26]. Whether this transient increase in plasma leptin is related to changes in leptin transport, metabolism or clearance is not known.

Plasma sampling in our study tend to correspond to ages when milk intake is the exclusive or dominant source of nutrient, namely at 2 and 4 months, and when mixed diet becomes increasingly established at 8 and 12 months respectively. Our preliminary data suggest that usual dietary intake in healthy and normally grown infants probably does not affect plasma leptin or its soluble receptor concentrations, although determination of the relationship between leptin and its receptor with details of nutritional intake was not the primary goal of this study and further studies are needed.

Anthropometric and body composition measurements are related to and predictive of plasma leptin. This is consistent with other reports that plasma leptin is correlated with actual [26] or gain [25,26] in body weight, and actual [8,9] and changes [9] in body mass index, and with indirect indicators of body fat such as subcutaneous skinfold thicknesses [7,25,26]. Plasma leptin was also found to discriminate both the long term and changes in energy status based on skinfold thickness [9].

The consistent relation between the plasma leptin particularly with fat mass is supportive of adipose tissue being the major source of circulating leptin. The negative correlation of percent bone mass and percent lean mass with plasma leptin is not surprising since an increase in the proportion of fat mass is generally correlated with decreased proportion of lean and bone mass. However, a direct relation between plasma leptin and other tissue mass may be possible since increasing numbers of non-adipose tissues including skeletal muscle [27], chondrocyte [28] and human osteoblast [29] are reported to synthesize leptin and may have cellular leptin receptor forms with physiological activity in experimental models. In any case, the exact role of leptin in the changes in skeletal muscle and bone in humans remain to be defined.

Our data show elevated sOB-R concentrations throughout infancy. Other investigators have reported persistently elevated sOB-R concentrations during early childhood [30]. The positive relation between plasma sOB-R and fat mass may be indicative of the increased membrane-bound leptin receptor forms, the source of sOB-R. The negative correlation of percent lean mass with plasma sOB-R is consistent with the generally inverse relation between percent lean and fat mass.

The correlation between these plasma variables with anthropometric or body composition measurements were better with the use of Z scores rather than absolute measurements, with fat percent rather than absolute fat mass, and generally better with body composition, specifically fat mass, rather than anthropometric measurements. Thus the use of standardized rather than absolute measurements of anthropometry and body composition is indicated in future studies on the interplay of leptin and its receptor with different nutrition support in growing subjects. Furthermore, body composition measurements are probably more sensitive indicators of leptin production and bioactivity.

It is interesting that even with the limited sample size, our findings of higher leptin concentration in African American infants independent of fat mass is consistent with the report on adult males and females that non-Hispanic blacks have slightly higher values compared to non-Hispanic whites or Mexican Americans [31]. Our data of higher plasma leptin concentration in female infants also are consistent with the presence of sexual dimorphism [9,32]. Furthermore, our data show that race and gender effects were eliminated in the presence of body composition measurements, presumably body composition measurements more specifically reflect the source of leptin and its receptors. Our data indicate that variations in plasma sOB-R are independent of race, gender or age but are predicted by body composition measurements.

Complexes of leptin with sOB-R reflect a molecular ratio of 1:1 [33] and reached a median value of >10 as early as 3 days after birth because of a decrease in circulating leptin with an accompanied increase in sOB-R [7]. Our data indicated that plasma sOB-R:leptin ratios remained >10 throughout infancy. Limited data indicate the high plasma sOB-R:leptin ratios may persist until 2 to 3 years [30]. It is possible that high circulating concentrations of sOB-R may block leptin function by its competition with the membrane receptor for the ligand, which in turn may be an important stimulus for energy uptake in the rapidly growing infant or in other conditions with a high energy demand. However, the decreasing sOB-R:leptin ratio during later infancy is presumably associated with increasing bioavailable leptin, and is consistent with slowing of growth [19] and tissue accretion [20-22].

Our report represents an exploratory step to determine the developmental variations of plasma leptin and its soluble receptor during the period of most rapid postnatal growth when the body weight and tissue accretion triples over a one year period. These data when coupled with the body composition measurements are critical to the design of future studies to determine the interplay of leptin and its receptors with nutrition support and the regulation of growth and tissue accretion.

## Conclusion

We conclude that in healthy growing infants, plasma leptin and sOB-R:leptin ratio but not sOB-R decreases with age. Body composition is the only variable that can predict plasma leptin and its soluble receptor and the receptor:ligand ratio, and body composition measurements eliminate the race and gender effect on these plasma variables. Based on limited size of subgroups in this study, the race and gender effect on these plasma variables appears to be consistent with that for adults.

## Competing interests

The author(s) declare that they have no competing interests.

## Authors' contributions

WK participated in design and execution of the study, analysis and interpretation of the data, and completion of the manuscript. MH participated in execution of the study, interpretation of the data, and manuscript writing. EH participated in statistical analysis, interpretation of the data and manuscript writing.
